# Opposing Immune Cell Responses to Viral Infections in Kidney Transplant Recipients: A Bibliometric Analysis

**DOI:** 10.1155/bmri/8096964

**Published:** 2025-11-29

**Authors:** Ruizhuang Sun, Shen Xu, Zhenjia Fan, Pu Li, Juping Zhao, Jun Meng

**Affiliations:** ^1^ Department of Laboratory Medicine, Ruijin-Hainan Hospital Shanghai Jiaotong University School of Medicine (Hainan Boao Research Hospital), Qionghai, China; ^2^ Department of Blood Transfusion, Ruijin Hospital, Shanghai Jiao Tong University School of Medicine, Shanghai, China, shsmu.edu.cn; ^3^ Department of Laboratory Medicine, Ruijin Hospital, Shanghai Jiaotong University School of Medicine, Shanghai, China, shsmu.edu.cn; ^4^ Department of Urology, Ruijin-Hainan Hospital Shanghai Jiaotong University School of Medicine (Hainan Boao Research Hospital), Qionghai, China

**Keywords:** BK polyomavirus, cytomegalovirus, immune response, immunosuppression, infection, kidney transplantation, NK cells, T cells

## Abstract

**Background:**

Infections remain a significant concern in kidney transplant recipients, affecting both graft and patient survival. Understanding the immune cell responses to various pathogens is essential for developing effective prevention and treatment strategies.

**Objective:**

The objective was to analyze research trends in kidney transplantation infection literature and characterize differential immune cell responses to common posttransplant infections.

**Methods:**

A comprehensive analysis of 4277 English language articles on kidney transplantation and infection from the Web of Science Core Collection was conducted. Research output, international collaboration, and keyword trends were analyzed. Immune cell responses to various infections in kidney transplant recipients were systematically evaluated.

**Results:**

The United States (3845 articles), France (1819 articles), and China (1342 articles) were the leading contributors to research in this field. Key research clusters included immunosuppression management, viral infections, and treatment strategies. Most significantly, analysis of immune cell populations revealed distinct patterns of response to different infections. Cytomegalovirus infection increased CD3 + CD8 + midCD56+ NK‐T cells and CD3 + CD8+ T cells, while BK polyomavirus reactivation decreased CD4+ and CD8+ T cells. Under immunosuppressive conditions, NK cell numbers were reduced. Kidney transplant infections directly caused decreases in CD4 + CD25+/CD4+ T cells, CD8 + CD25+/CD8+ T cells, and HLA‐DR+ monocytes, reflecting differential immune modulation based on infection type.

**Conclusion:**

Different pathogens elicit distinct immune cell responses in kidney transplant recipients, with some infections enhancing specific immune cell populations while others suppress them. These differential patterns of immune modulation reflect the complex interplay between immunosuppressive therapy and infectious agents. Understanding these specific immune responses provides valuable insights for developing targeted infection management strategies and improving monitoring protocols in transplant recipients.

## 1. Introduction

Kidney transplantation is a highly effective treatment for end‐stage renal disease, significantly improving both the survival rates and quality of life for patients [1, 2]. However, despite advancements in transplant surgical techniques, posttransplant infections remain a leading cause of mortality and graft failure [3, 4]. Kidney transplant recipients require long‐term immunosuppressive therapy to prevent graft rejection. While effective, these treatments also severely weaken the immune system, leaving patients highly susceptible to infections caused by bacteria, viruses, and fungi [5]. This immune vulnerability underscores the critical importance of effective infection management in the field of kidney transplantation [6, 7].

With continuous advancements in medical technologies, posttransplant infection management has evolved from focusing on acute phase treatments to adopting comprehensive, multidisciplinary approaches. These include strategies for infection prevention, immune regulation, and antibiotic resistance control. The introduction of immunosuppressive agents has altered the infection landscape, with viral infections, antibiotic resistance, and microbiota‐related complications becoming increasingly prominent research areas [8–10]. These developments not only reflect progress in kidney transplantation but also highlight the shift toward more personalized and precise treatment approaches in medical research.

To gain a deeper understanding of the research landscape surrounding posttransplant infections, this study employs bibliometric analysis to systematically examine the relevant literature. Bibliometrics, a quantitative method, enables the identification of research trends, key topics, and scientific advancements within a specific discipline. By analyzing metrics such as publication volume, citation frequencies, keyword co‐occurrence, and collaborative networks, this study is aimed at uncovering the current research status and future directions in the field of kidney transplantation and infection. Additionally, an analysis of international collaboration and academic influence provides a new perspective on this critical area of study.

For clinicians, researchers, and transplant recipients, understanding the current trends and priorities in kidney transplantation infection research is essential. Using bibliometric methodologies, this study highlights research hotspots, key developments, and emerging frontiers in the field, offering a valuable reference for clinical research and decision‐making in diagnosing and managing posttransplant infections.

## 2. Materials and Methods

In this study, the core collection of Web of Science (WOS) was used as the data source, and the search query was formulated as TOPIC = “Kidney transplantation” AND “infection”. The search was conducted using Boolean logic to combine the terms “Kidney transplantation” and “infection” in the topic field. A total of 4403 articles were retrieved. In the screening process, 123 non‐English articles were excluded, only 4280 English articles were retained, and three articles that had been removed from WOS core journals were further excluded. Finally, 4277 eligible articles were included as research objects.

The search was conducted on February 11, 2025, with the database update or access date reflecting this. The filtering criteria included document types such as research articles, reviews, and conference papers, while subject areas were not restricted. In order to deeply analyze the research status and development trend in this field, this study uses the bibliometric software Bibliometrix (Version 3.0) to process and analyze the data, mainly covering the following three aspects: first, the analysis of national output, statistics of the number of literature published in this research field in various countries to reveal the distribution of international cooperation and regional research; the second is the author keyword co‐occurrence analysis. By constructing a keyword co‐occurrence network, the correlation between keywords and high‐frequency research topics is discussed. Only keywords with frequency ≥ 10 are included to ensure the reliability and representativeness of the analysis results. The third is time trend analysis. Based on the annual distribution data of keywords, it shows the time evolution trend of research topics in this field. In addition, the research hotspots and dynamic development are presented intuitively through visualization tools (such as keyword network diagram and time evolution trend diagram). The method design of this study is aimed at revealing the key areas, core themes, and international cooperation patterns of “Kidney transplantation” and “infection”–related research and providing reference for future research in this field.

The definition of the “influence index” used in Bibliometrix was based on citation counts and the *h*‐index, allowing for the identification of influential articles in the field.

## 3. Results

### 3.1. Main Information Analysis

**Figure 1:**
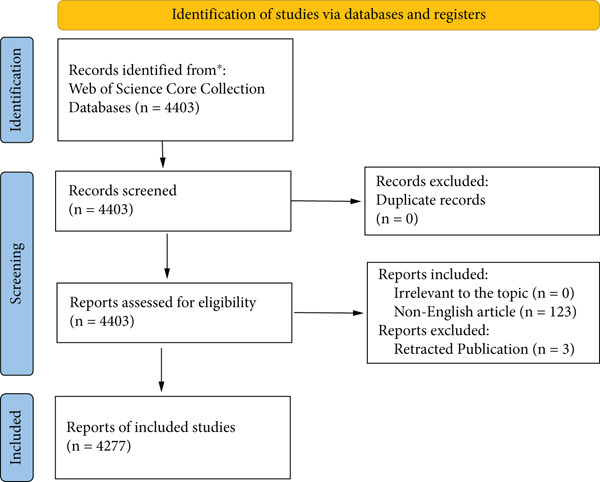
Article data included in the graph. Consider, if feasible to do so, reporting the number of records identified from each database or register searched (rather than the total number across all databases/registers). If automation tools were used, indicate how many records were excluded by a human and how many were excluded by automation tools.

As shown in Figure 1, after filtering the 4403 articles identified in the WOS Core Collection, 4277 English‐language articles on kidney transplantation and infection were included in the study. These articles were analyzed to identify citation patterns and key publication sources. See Figure 2a, the three most frequently cited journals were Transplantation (13,321 citations), American Journal of Transplantation (11,611 citations), and Transplant Proceedings (4062 citations). See Figure 2b, the American Journal of Transplantation ranked highest in impact with an influence index of 69, followed by Transplantation (57) and Nephrology Dialysis Transplantation (35). See Figure 2c, over the past 20 years, these journals have seen a consistent increase in publication output, with the most notable growth observed between 2000 and 2020. However, since 2020, the growth rate of the American Journal of Transplantation and Transplantation has slowed, reflecting stricter submission standards and a focus on innovative research areas.

Figure 2Bibliometric analysis of research impact and productivity. (a) Most locally cited sources. (b) Sources′ local impact. (c). Sources′ production over time. (d). Authors′ local impact. (e). Countries′ scientific production. (f). Countries′ scientific production.

**Figure:**
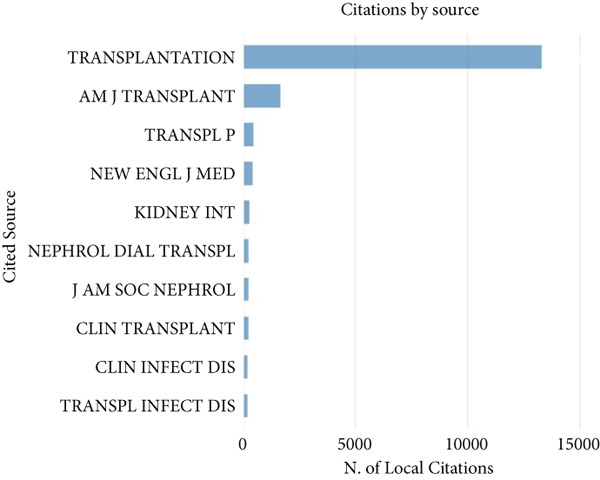
(a)

**Figure:**
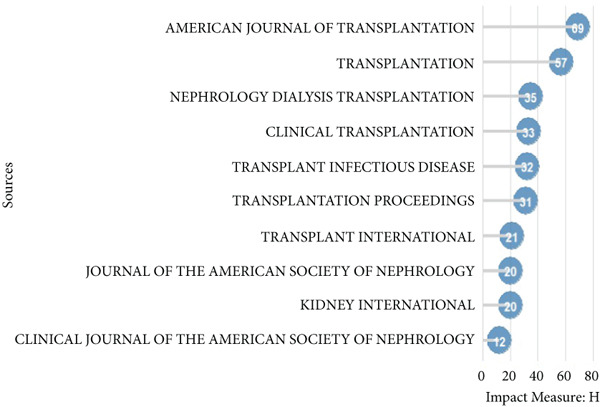
(b)

**Figure:**
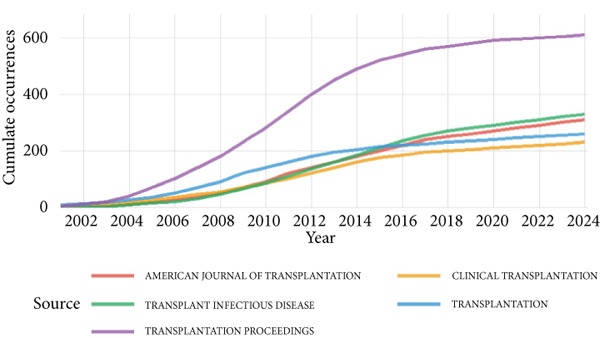
(c)

**Figure:**
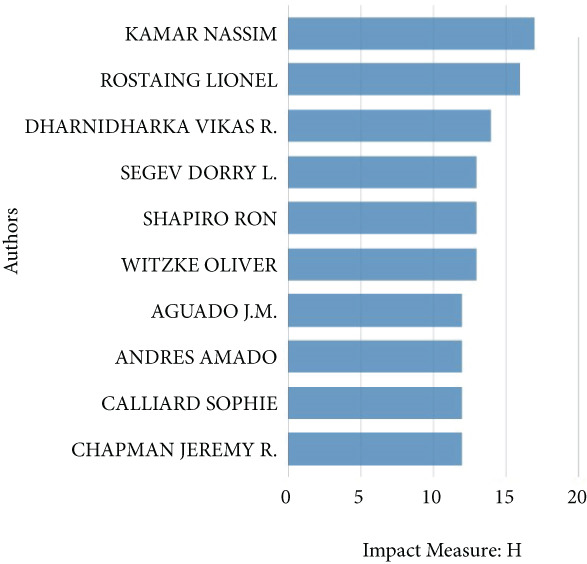
(d)

**Figure:**
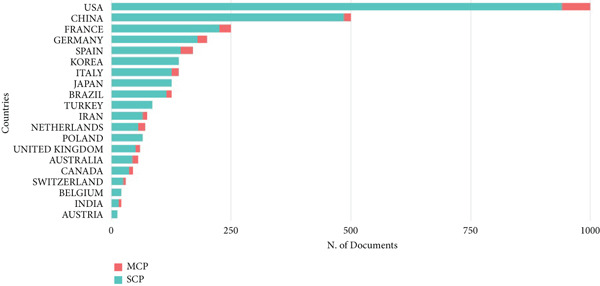
(e)

**Figure:**
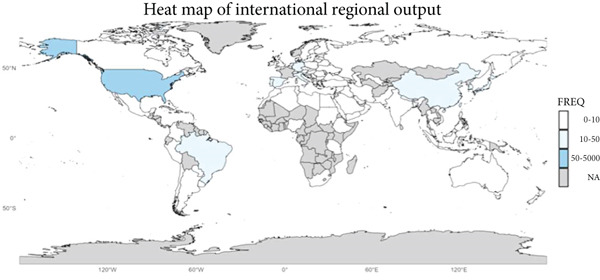
(f)

### 3.2. Scientific Output Analysis of Outstanding Authors and Countries

See Figure 2d, the analysis of author influence showed that Kamar Nassim had the highest impact (Influence Index 17), followed by Rostaing Lionel (Influence Index 15). (Figure 2e) Since 2015, the number of authors contributing to this field has grown significantly, indicating increasing research interest. The data also highlighted international collaboration patterns, with the United States leading in both total publications and co‐authored articles. Following the United States, France and China ranked second and third, respectively, in publication volume. See Figure 2f, the Top 5 contributing countries are the United States (3845 articles), France (1819 articles), China (1342 articles), Spain (1188 articles), and Germany (998 articles), reflecting global interest and collaboration in kidney transplantation and infection research.

### 3.3. Research Focus and Development Trends Based on High‐Frequency Keywords

See Figure 3a, keyword co‐occurrence analysis revealed several high‐frequency terms, including “recipients,” “disease,” “impact,” “risk factors,” “survival,” “therapy,” “outcomes,” “rejection,” and “management.” These keywords highlighted the primary focus on infection risk, prognosis, and the classification of infectious diseases in kidney transplant patients. See Figure 3b,d, the frequency of keywords such as “recipients,” “disease,” and “impact” showed a notable increase after 2018, reflecting growing research attention on patient outcomes and infection management. Keyword evolution analysis from 2000 to 2024 revealed a shift in research focus. Between 2000 and 2010, studies primarily addressed “rejection” and “acute infection,” while research from 2010 onward shifted to “immunosuppression,” “antibiotic resistance,” and “infection prevention.” More recently, keywords like “antibiotic stewardship,” “prevention strategies,” and “immune modulation” have emerged, signifying a growing emphasis on personalized and strategic approaches to managing infections.

Figure 3A comprehensive analysis of word frequency and topic evolution. (a) Most frequent words. (b) Words′ frequency over time. (c) Trend topics.

**Figure:**
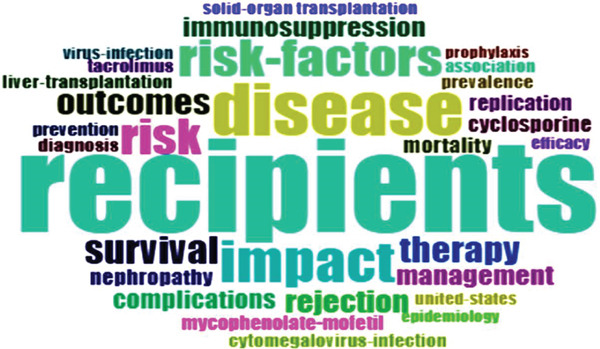
(a)

**Figure:**
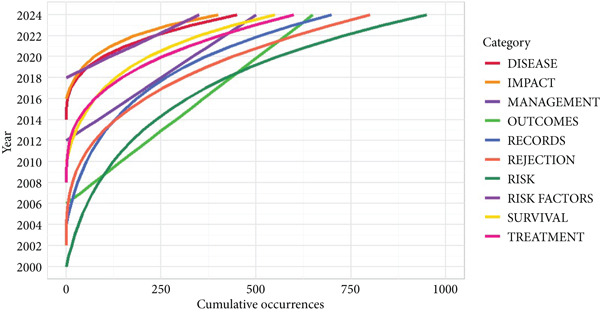
(b)

**Figure:**
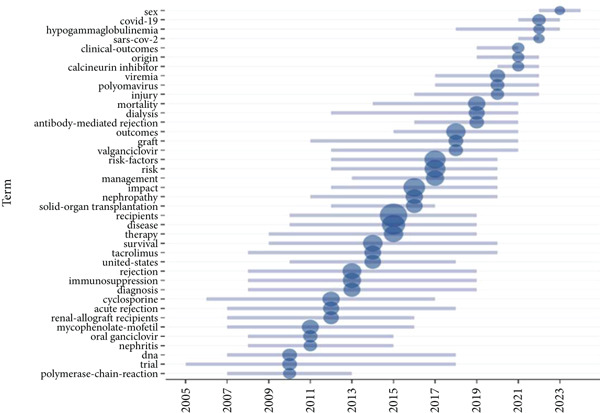
(c)

As the keyword evolution analysis highlighted the shifting focus from rejection and acute infection to immunosuppression and infection prevention, the next step is to examine the biological effects of these evolving research themes. Specifically, analysis of immune cell populations in kidney transplant recipients revealed varying effects of different infections on immune cells. As shown in Table 1, cytomegalovirus (CMV) infection led to increased numbers (+) of CD3 + CD8 + midCD56+ NK‐T cells and CD3 + CD8+ T cells. In contrast, BK polyomavirus reactivation resulted in decreased numbers (−) of CD4+ T cells, with observations of a decline in both CD4+ and CD8+ T cells (−). Under immunosuppressive conditions, NK cell numbers were reduced (−). Additionally, kidney transplant infections directly caused decreases (−) in CD4 + CD25+/CD4+ T cells, CD8 + CD25+/CD8+ T cells, and HLA‐DR+ monocytes. The results indicate that different types of infections have varied effects on immune cell populations in kidney transplant recipients, with some infections such as CMV increasing specific immune cell numbers, while others like BK polyomavirus decreasing specific immune cell counts. These immune cell changes reflect the complex immune status in posttransplant patients and provide a basis for clinical monitoring.

**Table 1:** Impact of various infections on immune cell populations in kidney transplantation.

**Infection type**	**Immune cell population**	**Effect**	**Journal (PMID)**
Cytomegalovirus infection	CD3 + CD8 + midCD56+ NK‐T cells	Increase (+)	Kidney Int (PMID: 38685562)
Cytomegalovirus infection	CD3 + CD8+ T cells	Increase (+)	Kidney Int (PMID: 38868358)
Immunosuppression	NK cells	Decrease (‐)	Front Immunol (PMID: 32793200)
Polyomavirus BK reactivation	CD4+ T cells	Decrease (‐)	Iran J Allergy Asthma Immunol (PMID: 37767680)
Polyomavirus BK reactivation	CD4+ and CD8+ T cells	Decrease (‐)	Clin Transl Immunology (PMID: 31956413)
Kidney transplant infection	CD4 + CD25+/CD4+ T cells, CD8 + CD25+/CD8+ T cells, and HLA‐DR+ monocytes	Decrease (‐)	Observational Study (PMID: 26554788)

**Figure 4:**
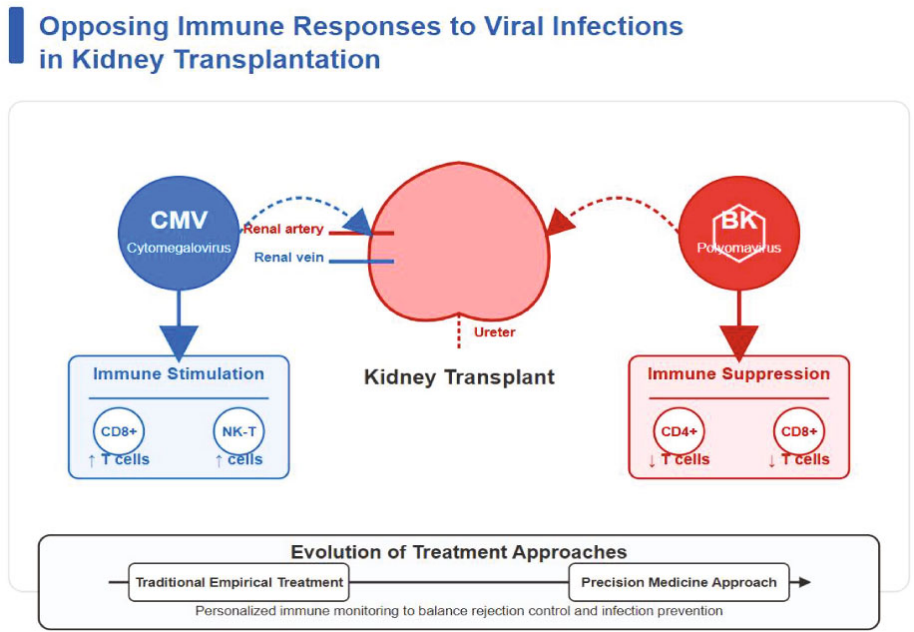
Opposing immune cell responses to viral infections in kidney transplantation.

In light of these findings, the immune response patterns post‐transplantation vary significantly based on the type of viral infection. For instance, CMV infection promotes an increase in the number of CD8+ T cells and NK‐T cells, producing an immunostimulatory effect, while BK polyomavirus reactivation leads to a decrease in the number of CD4+ and CD8+ T cells, resulting in an immunosuppressive effect. This opposing immunomodulatory model has driven the evolution of treatment methods from traditional empirical treatment to precision medical approaches that emphasize individualized immune monitoring to balance rejection control and infection prevention (Figure 4).

### 3.4. Main Research Directions in the Field of Kidney Transplantation and Infection

See Figure 5a, keyword co‐occurrence networks identified several key research clusters: (1) immunosuppression and infection management, focusing on infection prevention, post‐transplant infection, and antibiotic resistance; (2) viral infection and immune response, emphasizing CMV infection and immune modulation; (3) microbiome and personalized treatment, exploring the role of microbiota and the application of personalized medicine; (4) risk factors and clinical management, addressing survival, long‐term outcomes, and risk assessment; and (5) treatment strategy and infection assessment, balancing immunosuppressive therapies with rational antibiotic use. High‐weight keywords such as “infection prevention,” “CMV infection,” and “antibiotic resistance” highlighted these as core research areas, while emerging terms like “fungal infections” and “personalized medicine” indicated potential future trends in the field.

Figure 5Co‐occurrence networks and thematic analysis. (a) Co‐occurrence network. (b) Thematic map.

**Figure:**
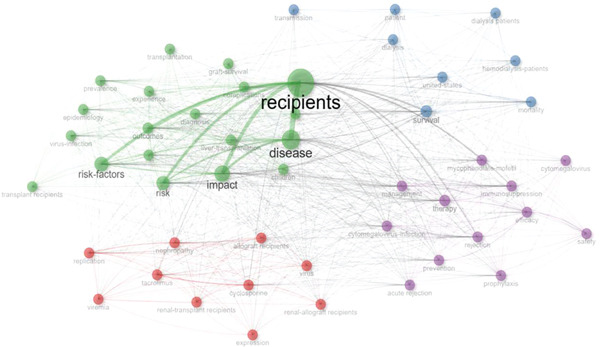
(a)

**Figure:**
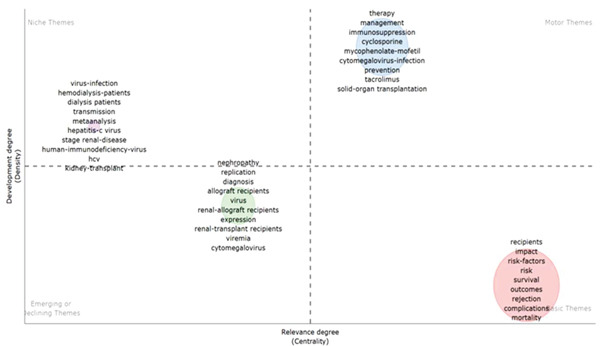
(b)

### 3.5. Topic Distribution and Evolution in Kidney Transplantation and Infection Research

Thematic mapping of research topics divided keywords into four quadrants based on centrality and density. Keywords like “infection prevention,” “immunosuppression,” and “antibiotic resistance” were identified as “motor themes,” signifying their central importance and strong development momentum. In contrast, terms such as “immune response” and “diagnostic biomarkers” were categorized as “basic themes,” providing foundational methodological support for the field. This thematic analysis demonstrated a clear evolution from traditional empirical approaches to advanced, data‐driven, and personalized infection management strategies, see Figure 5b.

## 4. Discussions

This study conducted an in‐depth analysis of academic research trends and future development in the field of kidney transplantation and infection using bibliometric methods. By analyzing 4277 academic articles, this study highlights key issues in infection management after kidney transplantation, particularly the progress and challenges in immunosuppression, antibiotic resistance, and viral infection control. Based on these findings, this discussion explores the evolution of infection research, the rise of personalized treatments, the balance between immune regulation and antibiotic management, and the application of global collaboration and new technologies.

According to bibliometric analysis, research on infections after kidney transplantation has shown a significant upward trend since 2000, with a notable acceleration in the past decade. The focus has shifted from managing rejection to the comprehensive management of infections. The widespread use of immunosuppressive therapies, while effective in preventing rejection, has significantly suppressed the immune system of renal transplant recipients, increasing the risk of infection complications. This shift reflects a growing understanding of the complexity of infection management and the transition from acute infection treatments to long‐term, personalized management strategies.

Keyword co‐occurrence analysis identified several core areas of focus, including immunosuppression, antibiotic resistance, viral infections (e.g., CMV), and the microbiome. Citation analysis highlights the leading role of the United States in this field, particularly in combining immunosuppressive strategies with infection control and applying new technologies. Early studies primarily addressed hyperacute and acute rejection prevention using large‐scale clinical interventions [11, 12]. However, with the standardization of immunosuppressive drug use, research has shifted towards managing infections within the context of immunosuppression. This includes integrating immune monitoring, pathogen surveillance, and personalized treatment plans [4, 13–15]. For instance, studies have increasingly focused on dynamically adjusting immunosuppressive drug regimens based on individual immune profiles and infection risks to minimize excessive immune suppression and reduce infection incidence [16]. In‐depth analysis of immune cell populations in kidney transplant recipients has revealed differential effects of various infections on immune cells, providing important evidence for optimizing individualized treatment strategies [17]. CMV infection leads to increased numbers of CD3+CD8+midCD56+ NK‐T cells (PMID: 38685562) and CD3+CD8+ T cells (PMID: 38868358), and this expansion of cytotoxic T cells likely reflects the activation of specific immune defense mechanisms against viral infection [18, 19]. As a persistent latent virus, CMV can induce the differentiation and expansion of memory T cells, generating virus‐specific immune responses [20]. This phenomenon is particularly important in immunosuppressive environments, representing a key antiviral defense capability preserved by the host. In contrast, BK polyomavirus reactivation results in decreased CD4+ and CD8+ T cell counts (PMID: 31956413) [21]. This differential impact may be related to the virus′s immune evasion mechanisms, as BK virus may suppress host antiviral immune responses by interfering with T cell function or inducing T cell apoptosis [22–24]. Research indicates that under immunosuppressive conditions, NK cell numbers are reduced, potentially further weakening the innate immune defense against opportunistic infections in transplant recipients (PMID: 32793200) [25]. Additionally, kidney transplant infections directly cause decreases in CD4+CD25+/CD4+ T cells, CD8+CD25+/CD8+ T cells, and HLA‐DR+ monocytes [26]. This broad immunosuppressive effect may result from the synergistic action of infection and immunosuppressive drugs [27]. These immune cell changes reflect the complex immune status of post‐transplant patients and provide a basis for clinical monitoring [18, 25]. Understanding these specific immune response patterns helps develop more precise infection risk assessment tools and individualized immune regulation strategies. For example, by monitoring trends in specific T cell subsets, clinicians can identify infection types and assess immune function status early, allowing timely adjustment of immunosuppressant dosages to achieve a more precise balance between controlling rejection and preventing infection [28–30]. This personalized treatment approach based on immune cell characteristics represents a new direction in posttransplant infection management, integrating immune monitoring, pathogen detection, and therapeutic interventions into an integrated precision medicine strategy [31, 32].

## 5. Conclusion

This study analyzed 4277 articles on kidney transplantation infection using bibliometric methods. Since 2000, research has shifted from rejection management to comprehensive infection management, with the United States, France and China leading contributions. Five research clusters were identified, covering immunosuppression, viral infections, microbiome, risk factors, and treatment strategies. Key findings include the differential immune responses to infections: CMV increases specific T cell populations while BK polyomavirus decreases T cell counts. This research highlights the ongoing transition from empirical treatments to data‐driven precision medicine approaches based on immune cell characteristics, balancing rejection control with infection prevention.

## Disclosure

The publication of this paper is free from any undue influence or improper inducements. I affirm that the above statements are true and correct, and I am willing to bear legal responsibility for them.

## Conflicts of Interest

The authors declare no conflicts of interest.

## Author Contributions

Ruizhuang Sun, Shen Xu, and Zhenjia Fan contributed equally to this work.

## Funding

No funding was received for this manuscript.

## Data Availability

The processes of data acquisition and analysis in this study strictly adhere to academic standards and were not subject to any external interference.
